# The importance of the electrophysiological tests in the early diagnosis of ganglion cells and/or optic nerve dysfunction coexisting with pituitary adenoma: an overview

**DOI:** 10.1007/s10633-018-9659-5

**Published:** 2018-10-29

**Authors:** Ewelina Lachowicz, Wojciech Lubiński

**Affiliations:** II Department of Ophthalmology, Pomeranian Medical University, Powstańców Wlkp. Street, 72, 70-111 Szczecin, Poland

**Keywords:** Pituitary adenoma, Visual pathway dysfunction, PVEP, mfVEP, PERG

## Abstract

**Background and methods:**

Based on the available literature, it is suggested, in the clinical evaluation of the chiasmal tumors, that the following electrophysiological tests: visual evoked potentials to pattern-reversal stimulation, multifocal visual evoked potentials (mfVEPs), and pattern electroretinogram (PERG) play an important role in the diagnosis of the optic nerve and retinal dysfunction in the course of pituitary tumors.

**Results:**

Macroadenomas and also microadenomas may cause dysfunction of retinal ganglion cells (RGCs) and their axons, even in the absence of changes in the routine ophthalmological examination, retinal sensitivity in standard automated perimetry, and retinal nerve fiber layer thickness in optical coherent tomography. The most frequently observed changes in electrophysiological tests were as follows: in PVEPs—the crossed/uncrossed asymmetry distribution, altered waveform, increase in P100-wave peak time, and/or reduction in amplitude; in mfVEPs—the peak time prolongation and/or amplitude reduction in C1-wave; in PERG—the reduction in N95-wave amplitude and decreased N95:P50 amplitude ratio. Hemifield PVEPs were more often abnormal than full-field PVEPs. Multi-channel recording is recommended for the assessment of the anterior visual pathway. The use of mfVEP offers the possibility to register localized disturbances of the optic nerve and ganglion cells. Additionally, an amplitude of N95-wave reduction in PERG correlated with a lack of postoperative visual acuity recovery. The postoperative improvement in the visual field was found to be associated with a normal N95:P50 amplitude ratio. The RGCs dysfunction manifested by decrease in PhNR/b-wave amplitude ratio was associated with the worse visual fields outcome. A review of the literature summarizing the electrophysiological testing in the pituitary adenoma is discussed.

**Conclusion:**

In patients with pituitary tumor, detection of the early dysfunction of the visual pathway may lead to modification of the medical treatment regimen and reduce the incidence of irreversible optic nerve damage.

## Introduction

Chiasmal disorders may have an influence on the anterior visual pathway function, even in the early stage of the disease and can lead to optic nerve damage. The pituitary tumors are the most common cause of chiasmal syndrome, manifested as bitemporal hemianopia and associated symptoms [1]. It represents the most frequent intrasellar pathology and accounts for about 10–15% of all intracranial tumors [2, 3]. About 70% of pituitary adenomas occur in individuals aged 30–50 years [4].

Many studies’ results have suggested one or more genetic factors that may contribute to the formation of pituitary adenomas. Pituitary tumors are thought to result from a single cell mutation followed by clonal expansion involving dysregulation of cell growth through either activation of an unknown oncogene or inactivation of a tumor suppressor gene [5].

Pituitary adenomas are benign epithelial lesions that rarely metastasize [4]. The tumor is classified based on its size as microadenoma (dimension < 10 mm) and macroadenoma (dimension > 10 mm) [3, 4, 6]. Classification concerning endocrine function distinguishes hormone secreting from non-secreting tumors. Functional adenomas occur in 70% of cases, and prolactinomas are the most common secreting tumors and account for about 30%. Non-functional tumors comprise 25–30% of pituitary adenomas. The majority of non-secreting changes are macroadenomas which often grow to a significant size, causing mass effects [1].

### Growth rate

There are a few studies describing the natural history of the pituitary adenoma. The average tumor growth depends on the initial size, the hormonal activity, and the sex [7].

The mean tumor growth rate for the patients with pituitary adenoma was calculated to be 1147 ± 870 days, with functioning tumors demonstrating a significantly shorter growth rate at 747 ± 564 days compared with clinically non-functioning PA at 1334 ± 926 days. The mean growth rate based on histopathological subtypes was as follows: null cell—1579 ± 1235; gonadotrophs—1097 ± 668; prolactinomas—1222 ± 1223; somatotrophs—895 ± 612; corticotrophs—2215 ± 2098; and plurihormonal tumors—941 ± 502 days [8].

Enlargement of non-functioning adenomas without treatment occurs in about 10% of microadenomas and 23% of macroadenomas. Specifically, among the patients with microadenomas, 10% had tumor growth, 7% had reduction in tumor size, while 83% remained unchanged in size in follow-up magnetic resonance imaging (MRI) over an observation period of 1–8 years. In contrast, of the patients with macroadenomas, 23% were demonstrated to have tumor enlargement, 12% showed evidence of tumor size reduction, and 65% remained stable in size during the follow-up period [9].

Studies examining the natural history of untreated microprolactinomas have shown that significant growth of these tumors is uncommon. Six series of patients with microadenomas who were found to have computed tomography or tomographic evidence of prolactinomas were observed without treatment for a period up to 8 years. Of women with small adenoma, only 7% microprolactinomas had evidence for growth and demands therapy [10–12].

### Mechanism

The mechanism for visual dysfunction produced by the pituitary tumors is unclear. The most likely causes are either a direct compression of visual fibers and/or interference with their vasculature. The sella turcica with its resistant dural diaphragm constitutes a non-expansible cavity, which, upon invasion by a space-occupying lesion, generates an intrasellar hypertension. It is worth to know that this pressure must be greater than venous pressure for the production of changes in the sanguine return, greater than arterial pressure to block the blood flow [13–16]. In proportion to the size of the sella turcica, an occupying lesion only has to be of some fraction of a millimeter for the initiation of this mechanism. If it is of sufficient size to act mechanically on the cerebrospinal fluid circulation area, it will produce ischemia and affect the optic chiasm, and if it is large enough to compress the vessels, it will act more directly. It was proven that tumors greater than 1 mm cause distortion of the vessels of the inferior group to a greater degree than the compression caused by the nervous structures, thus generating ischemic phenomenon on the visual pathway [15]. The anatomical relationship between the optic chiasm and the pituitary gland also makes the optic pathway susceptible to influence by lesions expanding from the sella turcica. This condition can disrupt neural conduction along the axons, impairs anterograde and retrograde axoplasmic flow, and can be a cause of demyelination. Gradual chiasmal compression can increase the nerve fiber loss and retrograde degeneration of RGCs [17, 18]. Other possibilities of the visual pathway abnormalities are changes in metabolites, trophic factors, or proteases associated with developing neoplasm in the immediate microenvironment [19–21].

### Symptoms

The clinical symptoms of pituitary adenoma include hormonal and visual changes. The neuro-ophthalmological symptoms are mostly observed in the course of a non-functional tumor (70%). Approximately in one-third of patients with clinically apparent pituitary tumor, the visual disturbance occurs, which usually develops gradually and may not be noticed because of the adaptation process [1, 3, 4].

The main neuro-ophthalmological finding in pituitary tumors is visual field defect (50–96%). The classic abnormal perimetry result is the bitemporal hemianopia due to the growth of the tumor, which compresses the crossed nasal fibers in the chiasm [5, 18, 19, 22]. However, thirty percent of the patients had no visual field abnormalities because of tumor size, location, and/or growth direction [3, 18, 20, 23]. Additionally, a short duration or the slight compression of the optic chiasm cannot provide visual field defects, which typically appear when at least 30–50% of RGCs are damaged [20, 22].

The pituitary adenoma compressing the visual pathway can be also a cause of abnormal color vision (56%), visual acuity reduction (36–46%), and optic atrophy (31%). Papilledema, ocular motor palsies, and disturbance of the papillary reflexes are seldom (1.5–2%) [3, 4, 6, 18, 22, 24, 25].

### Management

The endocrine tests and neuroradiology are the investigations of choice in the patients with the pituitary adenoma. The ophthalmological examinations are applied as a complementary test in diagnosis of the intracranial tumors.

Regarding the follow-up of adenomas not meeting the criteria for surgery, the following tests should be performed: MRI at 1 year for microadenomas, at 6 months for macroadenomas to assess progression, and then less frequently if unchanged in size [26]. Visual field testing is recommended for patients with lesions abutting or compressing the optic nerves or chiasm at the initial evaluation and during follow-up (6 months and yearly) and endocrine testing for macroadenomas (6 months and yearly) [26, 27].

In general, treatment is indicated if mass effect from the tumor and/or significant effects from hyperprolactinemia are present [28]. Bromocriptine, as a dopamine agonist, can be a choice in the treatment of prolactinoma. It decreases both the synthesis and secretion of prolactin. Bromocriptine treatment leads to tumor reduction in approximately 80% of microprolactinoma and in 70% of macroprolactinoma of non-pregnant patients. Up to 70% of patients who do not respond to bromocriptine respond to cabergoline, which is better tolerated than bromocriptine [29]. Larger tumors, resistant to medical therapy, can be surgically excised or treated by radiotherapy [30].

Long-term studies show that during pregnancy, the pituitary gland grows in size, which leads to an increase in prolactin production. Data in the literature indicate that the risk of tumor enlargement during pregnancy may occur in 3% of those with microadenomas, 32% of those with macroadenomas that were not previously operated on, and 4.8% of those with macroadenomas with prior ablative treatment [31]. The likelihood of progression from microprolactinoma to macroprolactinoma ranges from 0 to 12.5% [32].

The risk of significant asymptomatic tumor widening during pregnancy is 4.5%, and that of symptomatic tumor growth is < 2% [31]. The risk of new neurological sequel development (headaches, neuropathy, stalk compression) during pregnancy ranges from 1.6 to 5.5% [30, 32]. The risk of developing visual loss during single or multiple pregnancies in patients with microadenomas was small [33]. In pregnant women with microprolactinomas, dopamine agonists can be stopped at the 8th week of gestation safely due to the low risk of clinically relevant tumor expansion [30, 34]. So patients should be followed up monthly according to clinical symptoms such as headache and visual disturbance. Visual field examinations should be done at baseline at the time of diagnosis and should be followed every trimester [31, 35].

The rate of all adverse outcomes in pregnant women with macroprolactinoma is 36%: headaches (99.6%), headaches and visual impairment (25%), and diabetes insipidus (< 1%). Subsequent studies have reported neurological symptoms (13%) and visual impairment (75%) in women with macroprolactinomas during pregnancy [32]. The first option is to discontinue the dopamine agonist after confirmation of pregnancy with close follow-up. Monitoring should include screening for symptoms such as headache and visual problems, visual field examination every 2 months, and pituitary MRI without a contrast agent after the first trimester, which can be individualized for each patient. Follow-up may be preferable in patients with relatively small macroadenomas away from the optic chiasma. The second option is continuing dopamine agonist therapy throughout pregnancy. It may be preferred when the duration of dopamine agonist therapy before conception is short or when the tumor is outside intrasellar boundaries. If clinical signs of progression such as severe headache and visual field defects occur, an MRI without gadolin should be performed, and then, a dopamine agonist should be restarted if there is an increase in tumor size. If there is no response to dopamine agonist therapy, delivery may be the treatment of choice when the term is close. Transsphenoidal surgery can be performed on patients whose term is not close [35, 36]. However, if there is rapid worsening of neurological sign and symptoms, it may be necessary to use a decompression surgery [30, 34].

In the current algorithm for pituitary tumor management, ophthalmological indications for surgery are as follows: a visual field deficit due to the lesion, other visual abnormalities, such as ophthalmoplegia or neurological compromise due to compression by the lesion, lesion abutting or compressing the optic nerves or chiasm on MRI, pituitary apoplexy with visual disturbance [27, 37].

### Electrophysiology

The previous data suggested that the electrophysiological tests (PVEPs, mfVEPs, PERG) can be a reliable method for assessing the visual pathway in patients with pituitary adenoma. Electrophysiological examinations are objective, sensitive, and noninvasive and can provide important information on the functional status of the visual system. These tests represent an extension of clinical examination, but are not yet routinely performed [23, 24, 38, 39].

In patients with pituitary adenoma, the above-mentioned tests may be useful in the following conditions: the initial assessment and the early diagnosis of the patient with visual symptoms, the identification of the sites of the dysfunction, the estimation of the severity of the disease, the evaluation of the progression and management of patients with radiologically confirmed lesion, and the conduction of the postoperative monitoring [24, 25].

The electrophysiological tests can be used in assessing the subjects whose visual symptoms are unreliable, atypical, still minimal, or unaffected, especially in early stage of the asymptomatic or non-secreting tumors. It is worth knowing the abnormalities in a visual pathway can be detected by electrophysiological tests before the appearance of structural changes, which can result in severe irreversible visual loss [6, 19, 20, 24, 40–43]. The electrophysiological examination should be used in the detection of early impairment of RGCs and/or optic nerve caused by pituitary adenoma, even without chiasmal compression seen in MRI. These tests can reveal abnormalities of a more widespread and insidious lesion of the visual pathway than expected based on tumor size [20]. The abnormalities of the electrophysiological examinations can be found even in patients without clinical evidence of the visual pathway impairment in visual acuity, perimetry, or optical coherent tomography (OCT) [6]. These recorded disturbances may be a first indicator of functional involvement of the chiasm and may influence the treatment strategies of the pituitary adenoma [24]. In patients with pituitary tumor, the early detection of the visual system abnormalities by electrophysiological tests and prompt treatment may be a cause of the reversible dysfunction of the visual pathway [20].

The aim of this paper is to present the current stage of knowledge concerning the visual electrophysiology in management of pituitary tumors.

### Visual evoked potentials

From the available literature, it may be concluded that in patients with chiasmal compressive lesions, most frequently VEPs were used. The full-field pattern-reversal VEPs are objective, quantitative, and repeatable tests and are more sensitive than conventional clinical examinations of visual acuity and visual fields [38, 44].

**Fig. 1:**
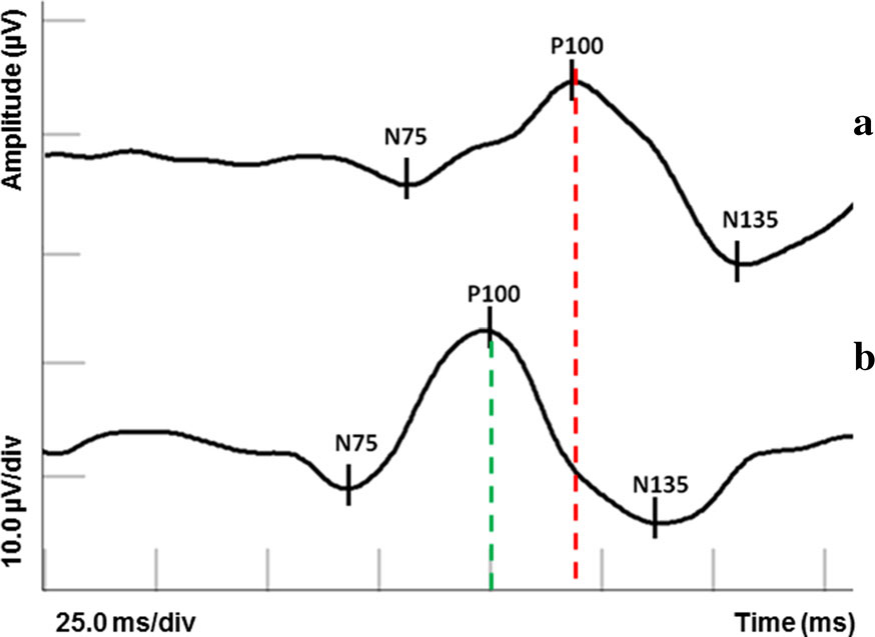
Comparison of the PVEP result in a patient with pituitary adenoma (**a**) versus the healthy individual (**b**)

Compression of the optic chiasm produces VEP asymmetries (24%) or results in an altered waveform (40%), amplitude reduction (41%), and peak time increase (34%, 1–32 ms) of P100-wave [38, 43, 45–47] (Fig. 1).

The amplitude changes may reflect in the conduction block in damaged fibers, whereas the delay of peak time may reflect in the slowed conduction through demyelinated segments of the nerve fibers, which occur as a result of compression [43, 46, 47].

**Fig. 2:**
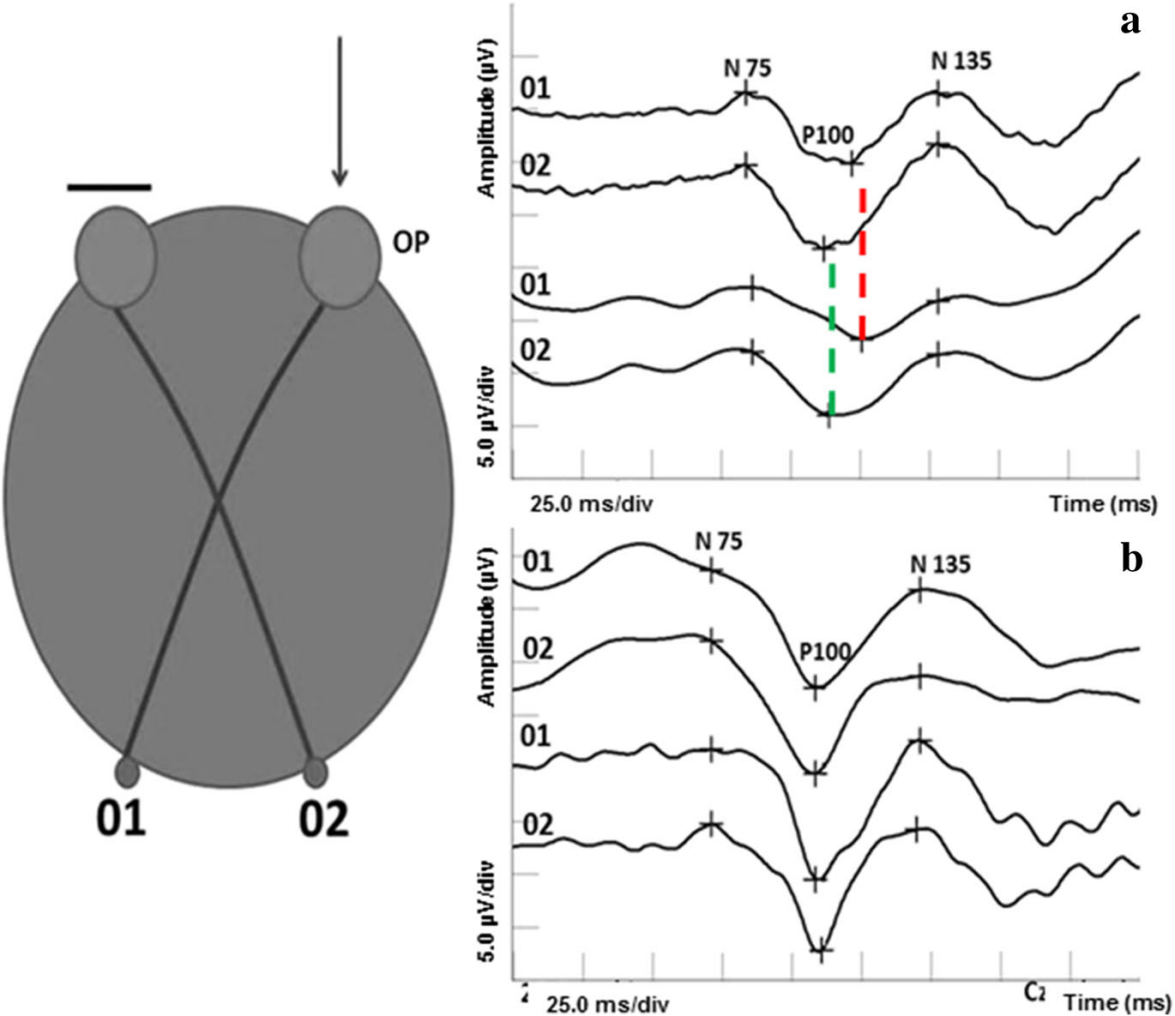
Comparison of the multi-channel VEP result in a patient with pituitary adenoma (**a**) versus the healthy individual (**b**)

If a tumor at the chiasm compresses the crossed optic nerve fibers from both eyes, the VEPs show a characteristic crossed asymmetry distribution (Fig. 2).

Chiasmal dysfunction gives a crossed asymmetry, whereby the lateral asymmetry obtained on the stimulation of one eye is reversed when the other eye is stimulated. The pattern stimuli should be presented with a field of 30 degree. A minimum of two channels are needed to detect lateral asymmetries. The minimum of three active electrodes (two lateral electrodes placed at O1 and O2 and a third midline active electrode at Oz) referenced to Fz should be used. It correlates with temporal field defects and bitemporal hemianopsia. Particular caution is needed when interpreting multi-channel pattern-reversal VEPs because of the paradoxical lateralization. This phenomenon, in which the response recorded at a lateral scalp location is generated by activity in the contralateral hemisphere of the brain, occurs with a large field, large check reversal stimulus, and common reference recording to Fz [48]. Bipolar recordings using ipsilateral hemisphere reference electrodes do not show paradoxical lateralization of the signal recorded via Fz reference is thus apparent [24]. When the tumor is more posterior and lateral to one side of the optic chiasm, the VEPs can show an uncrossed asymmetry distribution [23, 38, 44–47]. Multi-channel VEP recording is not required for a basic ISCEV standard clinical VEP. However, assessment of the chiasmal and postchiasmal visual pathway dysfunction requires multi-channel recording for accurate diagnosis [48].

The PVEPs often indicated marked functional lack of symmetry, whereas the results of the neuroradiology suggested regular midline suprasellar extension [44].

Several studies’ results have demonstrated that half-field stimulation reveals abnormalities not detectable with full-field stimulation in chiasmal lesions, but adequate consideration of registration parameters is critical to VEP interpretation [44]. Hemifield stimulation has shown greater sensitivity (36–85%) than full-field stimulation (29–74%) of early chiasmal involvement [23, 38, 43–46]. This is due to a separated registration of conduction along the crossed and uncrossed fibers in half-field PVEPs and corresponds to the isolated damage, while recording in full-field can be normal because it is compensated by the conduction of impulses through the undamaged fibers [43–46, 49, 50]. Some patients with reduced visual acuity have difficulty maintaining accurate fixation, and it might not be possible to perform hemifield stimulation in some cases [44].

### Multifocal visual evoked potentials

The multifocal visual evoked potential may also be used in diagnosis of compressive optic neuropathy [6, 51, 52]. This method is useful in detecting optic pathway dysfunction providing visual field loss. The mfVEPs give a map of RGCs and their axons usually from central 50°, and its result corresponded topographically with retinal sensitivity measured by static perimetry [6, 50, 53].

By using more than one channel to record the mfVEPs signals, the sensitivity of the method can be improved [52, 54, 55]. A method based on the analysis of the SNR (signal-to-noise ratio) of mfVEP signal amplitude improves the assessment of patients at risk of developing visual field defect. However, inter-individual differences and recording variability make the interpretation of this test technique sometimes difficult [55].

**Fig. 3:**
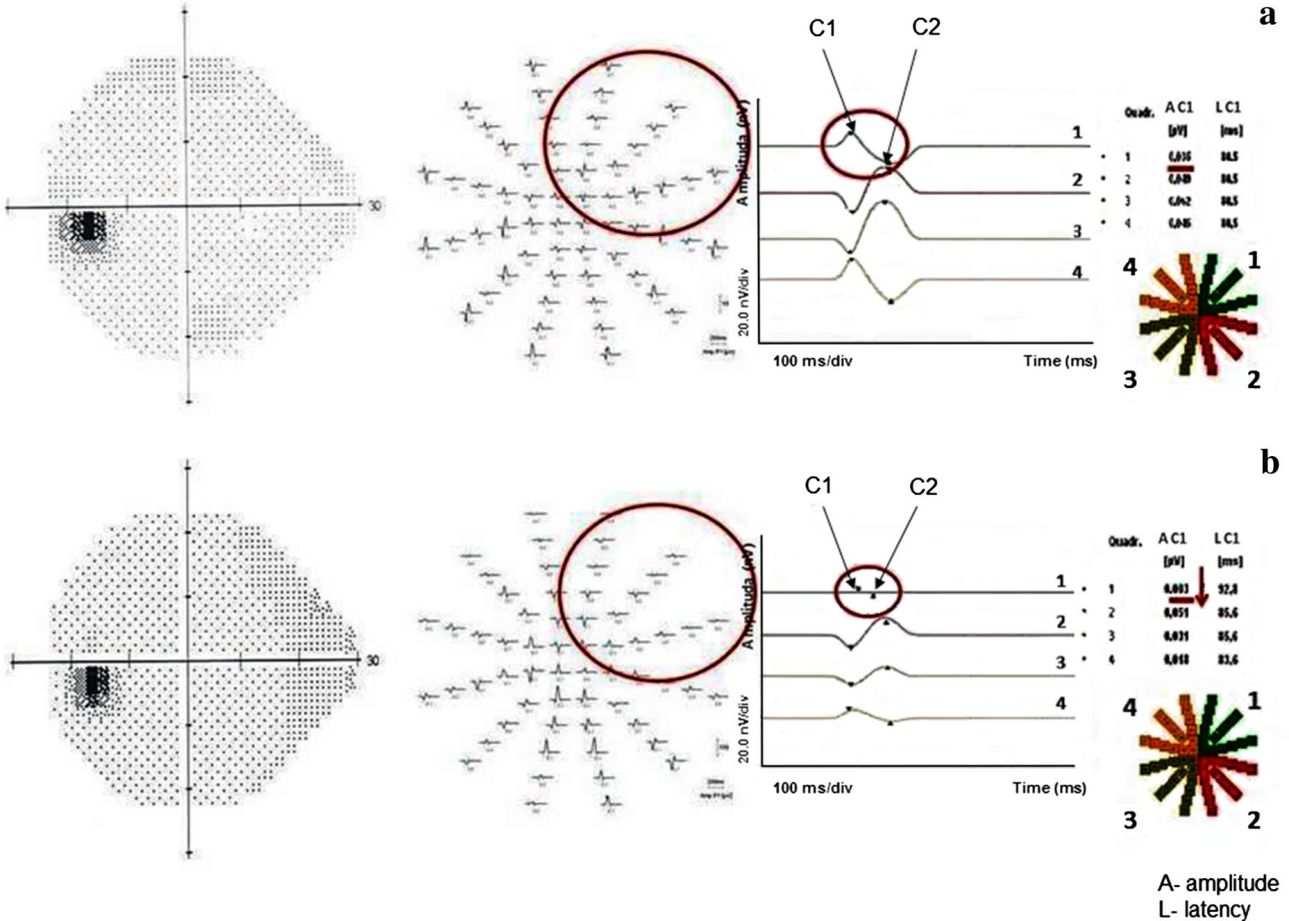
Comparison of the VF 24-2 (W-W) and mfVEP result in the healthy individual (**a**) versus a patient with pituitary adenoma (**b**)

Chiasmal compression due to pituitary adenoma causes the reduction in amplitudes and increase in peak time of the mfVEPs response (Fig. 3).

Abnormality in mfVEPs that are present mainly in the upper temporal quadrant can manifest as the amplitude reduction in C1-wave associated with damage of the retinal ganglion cells and/or their axons [6, 51, 53]. The superotemporal quadrants of vision are usually first affected in consequence of the inferonasal fibers compression [24, 41]. The increased C-wave peak time may be a first feature of localized optic nerve dysfunction, which was not otherwise apparent on conventional tests [6, 53].

Electrophysiological abnormalities located in another quadrant may be most likely caused by the intrasellar hypertension [14–16]. Additionally, this may have an importance in the significance of mfVEPs in early diagnosis and follow-up of patients with pituitary tumor.

The monitoring of the mfVEPs parameters may help to assess the progression of disease objectively, even in subject with normal visual field, due to small differences in the response between subsequent sessions in the same patient [51].

The mfVEPs, SAP, and OCT provided complementary information for detecting visual pathway abnormalities in patients with pituitary adenoma. Several studies’ results suggest functional tests (mfVEP and SAP) detected earlier and more abnormalities in patients with pituitary adenoma than the structural test, such as OCT [56]. Correlation between mfVEPs and OCT in patients with pituitary adenoma was limited by the fact that SAP and mfVEPs measure the function of the entire visual pathway, whereas OCT only measures retinal ganglion cells complex (GCC) and optic nerve integrity from smaller area [20].

### Pattern electroretinogram

**Fig. 4:**
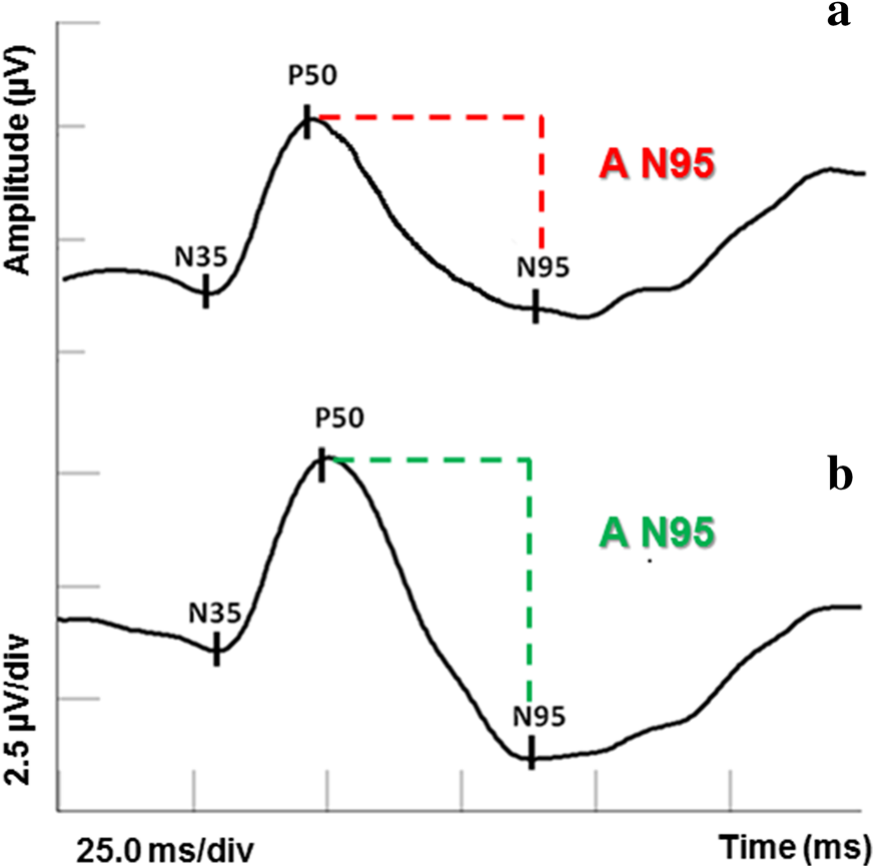
Comparison of the PERG result in a patient with pituitary tumor (**a**) versus the healthy individual (**b**)

The PERG is a next electrophysiological test, which can be useful in the early diagnosis and management of patients with chiasmal lesions [20, 23, 24]. The positive P50 component is invariably affected in retinal and macular dysfunction (19%), whereas the negative N95 component is generated (100%) from RGCs and is principally affected in optic nerve disease (81%) [44]. In more severe RGC damage, P50 may also show amplitude reduction (Fig. 4).

Furthermore, some diagnostic value has analyzed the ratio between N95 and P50 amplitudes [40, 41]. It has been suggested that N95/P50 ratio may be an important prognostic indicator for visual outcome in the preoperative assessment of optic nerve compression by the pituitary tumor. The postoperative worsening in the visual field was found to be associated with the abnormal PERGN95/P50 ratio. A disturbed PERG N95-wave correlates with a lack of postoperative visual acuity recovery, despite the successfully performed neurosurgical removal of the tumor [23, 24, 38, 40].

The improvement in visual function following the early diagnosis and timely appropriate intervention of the pituitary adenoma is highly variable and can be observed. It may be connected with reversible short-lasting dysfunction of the anterior visual pathway. Probably, the reversible RGC dysfunction is due to improvement in axoplasmic transport and metabolic and ischemic insults that can act along the course of the axon after reducing the tumor mass [20, 40, 57].

In patients with chiasmal compression, PERG amplitude and OCT RNFL, and macular thickness measurements were significantly related to visual field loss, but not to each other. The study results suggest that the two technologies quantify neuronal loss differently in patients with chiasmal compression and are complementary tests [58, 59].

### Photopic negative response

The photopic negative response (PhNR) is a negative wave that follows the photopic b-wave and can be used for evaluating the degree of damages in RGCs in patients with compressive optic neuropathy [60–62]. The PhNR has prognostic value in the preoperative assessment of chiasmal compression and for predicting postoperative visual outcome. The decreased PhNR/b-wave amplitude ratio correlated significantly with the worse postoperative visual field (MD and temporal visual field sensitivity). Moreover, PhNR is relevant as a functional indicator compared with the structural OCT, which reflects structural changes. The PhNR has several practical advantages compared with PERG. It is not require refractive correction, clear ocular media, and exact foveal fixation because is elicited by a strong flash [63].

### Serial recordings

The electrophysiological tests can monitor dynamics of the functional state of the chiasm [23]. The serial tests are the method of monitoring optic nerve and chiasm deterioration in the course of the disease [49]. They may even detect visual pathway dysfunction prior to the disturbances observed in the other ophthalmological tests (visual acuity, static perimetry, and OCT) and thus influence the management of the pituitary tumor.

A patient with suspected influence of the pituitary adenoma on the chiasm should be sent to complete routine ocular diagnostic test and also neuroimaging (CT scanning and/or MRI). To detect the visual pathway disturbances concern with silent tumor growth, the authors suggested that additionally electrophysiological tests such as PERG and multi-channel PVEP (optionally mfVEP and PhNR) should be performed as follows: baseline and at 6 months for microadenomas to assess progression, at 3 months for macroadenomas, and then less frequently if unchanged in size. If the tumor compress the visual pathway the examinations should be performed more frequently.

## Conclusion

In the early stage of the optic nerve and RGCs dysfunction due to pituitary adenoma, the abnormalities of electrophysiological tests can be found even without clinical evidence of the visual impairment in the routine ophthalmological examination and additional tests such as perimetry or OCT. Early detection of chiasmal dysfunction by electrophysiological tests and prompt treatment of the condition can prevent optic nerve neuropathy and severe irreversible visual loss. The electrophysiological tests should be used in the detection of early impairment of ganglion cells and/or optic nerve caused by pituitary adenoma, even without evident chiasmal compression in MRI, as well as for assessment of the progression and management of patients.

## References

[1] Rennert J, Doerfler A (2007). Imaging of sellar and parasellar lesions. Clin Neurol Neurosurg.

[2] Asa SL, Ezzat S (1998). The cytogenesis and pathogenesis of pituitary adenomas. Endocr Rev.

[3] Anderson D, Faber P, Marcovitz S, Hardy J, Lorenzetti D (1983). Pituitary tumors and the ophthalmologist. Ophthalmology.

[4] Liu GT, Volpe NJ, Galetta SL (2010) Vision loss: disorders of the chiasm. In: Grant T, Liu MD, Volpe NJ, Galetta SL (eds) Neuro-ophthalmology: diagnosis and management, 2nd edn. Saunders/Elsevier, Philadelphia, 2(7):237–259

[5] Herse P (2014). Pituitary macroadenoma: a case report and review. Clin Exp Optom.

[6] Jayaraman M, Ambika S, Ganfhi RA, Bassi SR, Ravi P, Sen P (2010). Multifocal visual evoked potential recordings in compressive optic neuropathy secondary to pituitary adenoma. Doc Ophthalmol.

[7] Delgrange E, Trouillas J, Maiter D, Donckier J, Tourniaire J (1997). Sex-related difference in the growth of prolactinomas: a clinical and proliferation marker study. J Clin Endocrinol Metab.

[8] Monsalves E, Larjani S, Godoy BL, Juraschka K, Carvalho F, Kucharczyk W, Kulkarni A, Mete O, Gentili F, Ezzat S, Zadeh G (2014). Growth patterns of pituitary adenoma and histopathological correlates. JCEM.

[9] Huang W, Molitch ME (2018). Management of nonfunctioning pituitary adenomas (NFAs): observation. Pituitary.

[10] Gillam MP, Molitch ME, Lombardi G, Colao A (2006). Advances in the treatment of prolactinomas. Endocr Rev.

[11] Weiss MH, Teal J, Gott P, Wycoff R, Yadley R, Apuzzo ML, Giannotta SL, Kletzky O, March C (1983). Natural history of microprolactinomas: six-year follow-up. Neurosurgery.

[12] Schlechte J, Dolan K, Sherman B, Chapler F, Luciano A (1989). The natural history of untreated hyperprolactinemia: a prospective analysis. J Clin Endocrinol Metab.

[13] Madrazo-Navarro I, Maldonado-León JA (1991). Campimetric alterations caused by pituitary microadenoma successfully treated by transsphenoidal adenomectomy. Arch Invest Med (Mex).

[14] Medvedev IuA, Savost’ianov TF, Denikina OE (1997). Hypophysis compression syndrome in the sella turcica: mechanisms of development, pathology. Arkh Patol.

[15] Dawson BH (1958). The blood vessels of the human optic chiasma and their relation to those of the hypophysis and hypothalamus. Brain.

[16] Arafah BM, Prunty D, Ybarra J, Hlavin ML, Selman WR (2000). The dominant role of increased intrasellar pressure in the pathogenesis of hypopituitarism, hyperprolactinemia, and headaches in patients with pituitary adenomas. J Clin Endocrinol Metab.

[17] Morgan JE (2004). Circulation and axonal transport in the optic nerve. Eye (Lond).

[18] Cannavo S, De Natale R, Curto L, Li Calzi L, Trimarchi F (1992). Effectiveness of computer assisted perimetry in the follow-up of patient with pituitary microadenoma responsive to medical treatment. Clin Endocrinol.

[19] Gutowski NJ, Heron JR, Scase MO (1997). Early impairment of foveal magno- and parvocellular pathways in juxta chiasmal tumours. Vis Res.

[20] Ventura LM, Venzara FX, Porciatti V (2009). Reversible dysfunction of retinal ganglion cells in non-secreting pituitary tumors. Doc Ophthalmol.

[21] Cioffi GA (2005). Ischemic model of optic nerve injury. Trans Am Ophthalmol Soc.

[22] Hershenfeld SA, Sharpe JA (1993). Monocular temporal hemianopia. Br J Ophthalmol.

[23] Brecelj J (2014). Visual electrophysiology in the clinical evaluation of optic neuritis, chiasmal tumours, achiasmia, and ocular albinism: an overview. Doc Ophthalmol.

[24] Holder GE, Heckenlively JR, Arden GB (2006). Chiasmal and retrochiasmal lesions. Principles and practice of clinical electrophysiology of vision.

[25] Poon A, McNeill P, Harper A, O’Day J (1995). Patterns of visual loss associated with pituitary macroadenomas. Aust N Z J Ophthalmol.

[26] Donckier JE, Gustin T (2012). Pituitary incidentaloma: to operate or not to operate?. Acta Chir Belg.

[27] Freda PU, Beckers AM, Katznelson L, Molitch ME, Montori VM, Post KD, Vance ML (2011). Endocrine society. Pituitary incidentaloma: an endocrine society clinical practice guideline. J Clin Endocrinol Metab.

[28] Serri O (1994). Progress in the management of hyperprolactinemia. N Engl J Med.

[29] Colao A, Abs R, Bárcena DG, Chanson P, Paulus W, Kleinberg DL (2008). Pregnancy outcomes following cabergoline treatment: extended results from a 12-year observational study. Clin Endocrinol (Oxf).

[30] Molitch ME (2010). Prolactinomas and pregnancy. Clin Endocrinol (Oxf).

[31] Almalki MH, Alzahrani S, Alshahrani F, Alsherbeni S, Almoharib O, Aljohani N, Almagamsi A (2015). Managing prolactinomas during pregnancy. Front Endocrinol (Lausanne).

[32] Imran SA, Ur E, Clarke DB (2007). Managing prolactin-secreting adenomas during pregnancy. Can Fam Physician.

[33] Kupersmith MJ, Rosenberg C, Kleinberg D (1994). Visual loss in pregnant women with pituitary adenomas. Ann Intern Med.

[34] Witek P, Zielinski G (2013). Management of prolactinomas during pregnancy. Minerva Endocrinol.

[35] Karaca Z, Tanriverdi F, Unluhizarci Kelestimur F (2010). Pregnancy and pituitary disorders. Eur J Endocrinol.

[36] Melmed S, Casanueva FF, Hoffman AR, Kleinberg DL, Montori VM, Schlechte JA, Wass JAH (2011). Diagnosis and treatment of hyperprolactinemia: an endocrine society clinical practice guideline. JCEM.

[37] Melmed S, Colao A, Barkan A, Molitch M, Grossman AB, Kleinberg D, Clemmons D, Chanson P, Laws E, Schlechte J, Vance ML, Ho K, Giustina A, Acromegaly Consensus Group (2009). Guidelines for acromegaly management: an update. J Clin Endocrinol Metab.

[38] Brecej J (1994). Electrodiagnostics of chiasmal compressive lesions. Int J Psychophysiol.

[39] Holder GE (1997). The pattern electroretinogram in anterior visual pathway dysfunction and its relationship to the pattern visual evoked potential: a personal clinical review of 743 eyes. Eye.

[40] Parmar DN, Sofat A, Bowman R, Bartlett JR, Holder GE (2000). Visual prognostic value of the pattern electroretinogram in chiasmal compression. Br J Ophthalmol.

[41] Piekarska A, Lubiński W, Palacz O (2003). Znaczenie badań perymetrycznych i elektrofizjologicznych w diagnostyce guzów przysadki mózgowej. Okulistyka.

[42] Badiu C, Serbănescu A, Coculescu M (1996). Pattern visual evoked potentials represent an early index for the evolution of optic chiasma syndrome of tumoraletiology. Rom J Physiol.

[43] Piekarska A, Lubiński W, Palacz O, Wieliczko W, Pynka S, Szych Z, Karczewicz D (2005). Znaczenie badań okulistycznych w wykrywaniu neuropatii nerwu wzrokowego towarzyszącej guzom przysadki mózgowej. Klin Ocz.

[44] Holder GE, Bullock PR (1989). Visual evoked potentials in the assessment of patients with non-functioning chromophobe adenomas. J Neurol Neurosurg Psychiatry.

[45] Halliday AM, Halliday E, Kriss A, McDonald WI, Mushin J (1976). The pattern-evoked potential in compression of the anterior visual pathways. Brain.

[46] Halliday AM, Halliday L, Kriss A, McDonald WI, Mushin J (1976). Abnormalities of the pattern evoked potential in compression of the anterior visual pathways. Trans Ophthalmol Soc N Z.

[47] Pietrangeli A, Jandolo B, Occhipinti E, Carapella CM, Morace E (1991). The VEP in evaluation of pituitary tumors. Electromyogr Clin Neurophysiol.

[48] Odom JV, Bach M, Brigell M, Holder GE, McCulloch DLL, Mizota A, Tormene AP (2016). ISCEV standard for clinical visual evoked potentials. Doc Ophthalmol.

[49] Stark DJ, Lenton L (1981). Electrophysiological assessment of compressive lesions of anterior visual pathways. Aust J Ophthalmol.

[50] Danesh-Meyer HV, Caroll SC, Gaskin BJ, Gao A, Gamble GD (2006). Correlation of the multifocal visual evoked potential and standard automated perimetry in compressive optic neuropathy. Invest Ophthalmol Vis Sci.

[51] Semela L, Hedges TR, Vuong L (2007). Serial multifocal visual evoked potential recordings in compressive optic neuropathy. Ophthalmic Surg Lasers Imaging.

[52] Hood DC, Odel JG, Winn BJ (2003). The multifocal visual evoked potential. J Neuroophthalmol.

[53] Piekarska A, Lubiński W, Gosławski W, Wieliczko W, Syrenicz A, Olszowski T, Karczewicz D (2008). Ocena przydatności badania mfVEP w diagnostyce guzów przysadki mózgowej. Klin Ocz.

[54] Klistorner AI, Graham SL, Grigg J, Balachandran C (2005). Objective perimetry using the multifocal visual evoked potential in central visual pathway lesions. Br J Ophthalmol.

[55] Kaltwasser C, Horn FK, Kremers J, Juenemann A, Bergua A (2011). Objective visual field determination in forensic ophthalmology with an optimized 4-channel multifocal VEP perimetry system: a case report of a patient with retinitis pigmentosa. Doc Ophthalmol.

[56] Qiao N, Zhang Y, Ye Z, Shen M, Shou X, Wang Y, Li S, Wang M, Zhao Y (2015). Comparison of multifocal visual evoked potential, static automated perimetry, and optical coherence tomography findings for assessing visual pathways in patients with pituitary adenomas. Pituitary.

[57] Pojda-Wilczek D, Pojda SM, Hendryk S, Herba E, Zatorska B, Jochan K (2000). Visual system function in patients after surgery for intracranial tumors. Neurol Neurochir Pol.

[58] Monteiro ML, Cunha LP, Costa-Cunha LV, Maia OO, Oyamada MK (2009). Relationship between optical coherence tomography, pattern electroretinogram and automated perimetry in eyes with temporal hemianopia from chiasmal compression. Invest Ophthalmol Vis Sci.

[59] Cennamo G, Auriemma RS, Cardone D, Grasso LF, Velotti N, Simeoli C, Di Somma C, Pivonello R, Colao A, de Crecchio G (2015). Evaluation of the retinal nerve fibre layer and ganglion cell complex thickness in pituitary macroadenomas without optic chiasmal compression. Eye.

[60] Rangaswamy NV, Frishman LJ, Dorotheo EU, Schiffman JS, Bahrani HM, Tang RA (2004). Photopic ERGs in patients with optic neuropathies: comparison with primate ERGs after pharmacologic blockade of inner retina. Invest Ophthalmol Vis Sci.

[61] Gotoh Y, Machida S, Tazawa Y (2004). Selective loss of the photopic negative response in patients with optic nerve atrophy. Arch Ophthalmol.

[62] Tamada K, Machida S, Yokoyama D, Kurosaka D (2009). Photopic negative response of full-field and focal macular electroretinograms in patients with optic nerve atrophy. Jpn J Ophthalmol.

[63] Moon CH, Hwang SC, Kim BT, Ohn YH, Park TK (2011). Visual prognostic value of optical coherence tomography and photopic negative response in chiasmal compression. Invest Ophthalmol Vis Sci.

